# Data on genetic polymorphism of flax (*Linum usitatissimum* L.) pathogenic fungi of *Fusarium, Colletotrichum, Aureobasidium, Septoria*, and *Melampsora* genera

**DOI:** 10.1016/j.dib.2020.105710

**Published:** 2020-05-15

**Authors:** Roman O. Novakovskiy, Ekaterina M. Dvorianinova, Tatiana A. Rozhmina, Ludmila P. Kudryavtseva, Aleksey A. Gryzunov, Elena N. Pushkova, Liubov V. Povkhova, Anastasiya V. Snezhkina, George S. Krasnov, Anna V. Kudryavtseva, Nataliya V. Melnikova, Alexey A. Dmitriev

**Affiliations:** a Engelhardt Institute of Molecular Biology, Russian Academy of Sciences, Moscow 119991, Russia; b Moscow Institute of Physics and Technology, Dolgoprudny 141701, Russia; c Federal Research Center for Bast Fiber Crops, Torzhok 172002, Russia; d All-Russian Scientific Research Institute of Refrigeration Industry – branch of V.M. Gorbatov Federal Research Center for Food Systems of Russian Academy of Sciences, Moscow 127422, Russia

**Keywords:** Fungi, Phytopathogens, *Fusarium*, Genetic polymorphism, Deep sequencing, Amplicon sequencing, Flax, *Linum usitatissimum* L

## Abstract

Being a valuable agricultural plant, flax (*Linum usitatissimum* L.) is used for oil and fiber production. However, the cultivation of this agriculture faces an urgent problem of flax susceptibility to fungal diseases. The most destructive ones are caused by the representatives of *Fusarium, Colletotrichum, Aureobasidium, Septoria*, and *Melampsora* genera, reducing flax yields significantly. To combat such pathogens effectively, it is of high importance to assess their genetic diversity that can be used to develop molecular markers to distinguish fungal genera and species. Morphological analysis traditionally carried out for fungal identification requires a given amount of time and tends to be difficult. In the present work, we determined the DNA sequences that are frequently used for phylogenetic studies in fungi – internal transcribed spacer (ITS) and beta-tubulin (*tub2*), translation elongation factor 1-alpha (*tef1*), RNA polymerase II largest subunit (*RPB1*), RNA polymerase II second largest subunit (*RPB2*), and minichromosome maintenance protein (*MCM7*) genes – for 203 flax fungal pathogens of *Fusarium oxysporum, F. avenaceum, F. solani, F. sporotrichiella, F. moniliforme, F. culmorum, F. semitectum, F. gibbosum, Colletotrichum lini, Aureobasidium pullulans, Septoria linicola*, and *Melampsora lini* species. The sequencing was performed using the Illumina MiSeq platform with a 300+300 bp kit, and on average, about 2350 reads per sample were obtained that allows accurate identification of the genetic polymorphism. Raw data are stored at the Sequence Read Archive under the accession number PRJNA596387. The obtained data can be used for fungal phylogenetic studies and the development of a PCR-based test system for flax pathogen identification.

Specifications table

**Table:** 

Subject	Genetics
Specific subject area	Molecular Genetics, Agricultural Mycology
Type of data	Amplicon sequence data
How data were acquired	Illumina MiSeq, 300+300 bp paired-end reads
Data format	Raw sequence reads, fastq format
Parameters for data collection	DNA was extracted from 203 flax (*Linum usitatissimum* L.) fungal pathogen samples of *Fusarium oxysporum, F. avenaceum, F. solani, F. sporotrichiella, F. moniliforme, F. culmorum, F. semitectum, F. gibbosum, Colletotrichum lini, Aureobasidium pullulans, Septoria linicola*, and *Melampsora lini* species obtained from the phytopathogen collection of the Institute for Flax (Torzhok, Russia)
Description of data collection	Amplicon libraries of fragments of internal transcribed spacer (ITS) and beta-tubulin (*tub2*), translation elongation factor 1-alpha (*tef1*), RNA polymerase II largest subunit (*RPB1*), RNA polymerase II second largest subunit (*RPB2*), and minichromosome maintenance protein (*MCM7*) genes of flax fungal pathogens of *Fusarium, Colletotrichum, Aureobasidium, Septoria*, and *Melampsora* genera were prepared using two-step PCR and sequenced on Illumina MiSeq with a 600-cycle kit
Data source location	Institute for Flax, Torzhok, Russia
Data accessibility	One can access amplicon sequence data at the NCBI Sequence Read Archive (SRA) under the accession number PRJNA596387 (https://www.ncbi.nlm.nih.gov/sra/PRJNA596387)

## Value of the data

• The dataset could be actively used for the assessment of genetic diversity and phylogenetic investigations of fungi belonging to *Fusarium, Colletotrichum, Aureobasidium, Septoria*, and *Melampsora* genera.
• The data can support those working in the field of molecular genetics of fungi and those who work with plant fungal pathogens.
• The kinship and evolution of fungi of *Fusarium, Colletotrichum, Aureobasidium, Septoria*, and *Melampsora* genera could be estimated using the provided dataset.
• The generated data open up an opportunity to create a test system for the identification of flax pathogens basing on the information on genetic polymorphism.

## 1 Data Description

Being a source of oil and fiber [1], flax is affected by numerous fungal pathogens. The most serious diseases of this plant are caused by the representatives of such species as *Fusarium oxysporum* f. sp. *lini, F. avenaceum, F. culmorum, Melampsora lini, Colletotrichum lini, Septoria linicola*, and *Aureobasidium pullulans* [2,3]. Information on the genetic diversity of the listed species is lacking but is of high importance for the determination of their kinship and evolution, as well as for the development of effective ways to combat these flax pathogens.

For 203 samples of flax pathogens of *Fusarium, Colletotrichum, Aureobasidium, Septoria*, and *Melampsora* genera, we sequenced the fragments of the regions that are frequently used for phylogenetic studies in fungi: internal transcribed spacer (ITS) [4,5], beta-tubulin (*tub2*), translation elongation factor 1-alpha (*tef1*), RNA polymerase II largest subunit (*RPB1*), RNA polymerase II second largest subunit (*RPB2*), and minichromosome maintenance protein (*MCM7*) [6], [7], [8]. Amplicon libraries were prepared using two-stage PCR (primers for the first stage of PCR are listed in Supplementary Table S1) and deep sequenced on the Illumina MiSeq platform. On average, 2350 (range – 400-6000) paired-end reads (300+300 bp) per sample were obtained in the fastq format and deposited to the Sequence Read Archive (SRA) under the accession number PRJNA596387. These data play a key role in assessing the genetic diversity of flax fungal pathogens and the development of time-saving and accurate test systems for their identification. Basing on the given dataset, future experiments are likely to give further insight into the question of the kinship and evolution of fungi of the studied genera.

## 2 Experimental Design, Materials, and Methods

### 2.1 Material

203 samples of 12 fungal species were provided by the Institute for Flax (Torzhok, Russia) from their phytopathogen collection in 2018. Among them were *Fusarium oxysporum* (72 samples), *F. avenaceum* (23), *F. solani* (5), *F. sporotrichiella* (3), *F. moniliforme* (8), *F. culmorum* (5), *F. semitectum* (2), *F. gibbosum* (4), *Colletotrichum lini* (46), *Aureobasidium pullulans* (17), *Septoria linicola* (8), and *Melampsora lini* (10) species.

### 2.2 DNA extraction and quality control

DNA was extracted from fungal mycelium according to the standard CTAB protocol. Agarose gel electrophoresis (2% agarose) and the Qubit 2.0 fluorometer (Thermo Fisher Scientific, USA) were used to control DNA quality and evaluate DNA quantity.

### 2.3 DNA library preparation and sequencing

We amplified DNA fragments of 203 samples of fungi comprising ITS and *tub2, tef1, RPB1, RPB2*, and *MCM7* genes. Amplicon libraries were prepared according to the protocol [9] with two-stage PCR as we described earlier [10]. Amplification of target sequences using primers that comprised target-specific sequences [8,11] and overhang adapters was performed in the first step (Supplementary Table S1). For each sample, amplicons were then equimolarly pooled and the second PCR was performed with primers consisted of dual-index barcodes and sequencing adapters. Next, all PCR-products were equimolarly pooled and the quality of the library was evaluated by the 2100 Bioanalyzer (Agilent Technologies, USA), while the quantity – by the Qubit 2.0 fluorometer (Thermo Fisher Scientific). The library was sequenced using the MiSeq platform (Illumina, USA) and the Illumina MiSeq Reagent Kit v3 (2 × 300 bp reads).

## Appendix Supplementary materials

Appendix A. Supplementary data Image, application 1

Image, application 2
