# Supplementary material for: Data on genetic polymorphism of flax (Linum usitatissimum L.) pathogenic fungi of Fusarium, Colletotrichum, Aureobasidium, Septoria, and Melampsora genera
Source: Data Brief. 2020 May 15;31:105710. doi: 10.1016/j.dib.2020.105710 (PMC7256288; doi:10.1016/j.dib.2020.105710)
Supplement: Supplementary file 1 — Appendix A. Supplementary data [file mmc1.docx]

**Supplementary Table 1. Primers for the first stage of DNA library preparation**

| **Primer pair** | **Sequence** | **Reference** |
| --- | --- | --- |
| ITS_F  ITS_R | TCGTCGGCAGCGTCAGATGTGTATAAGAGACAGCTTGGTCATTTAGAGGAAGTAA  GTCTCGTGGGCTCGGAGATGTGTATAAGAGACAGTCCTCCGCTTATTGATATGC | [1] |
| tub2_F  tub2_R | TCGTCGGCAGCGTCAGATGTGTATAAGAGACAGGGTAACCAAATCGGTGCTGCTTTC  GTCTCGTGGGCTCGGAGATGTGTATAAGAGACAGACCCTCAGTGTAGTGACCCTTGGC | [2] |
| tef1_F  tef1_R | TCGTCGGCAGCGTCAGATGTGTATAAGAGACAGGAYTTCATCAAGAACATGAT  GTCTCGTGGGCTCGGAGATGTGTATAAGAGACAGGACGTTGAADCCRACRTTGTC | [3] |
| RPB1_F  RPB1_R | TCGTCGGCAGCGTCAGATGTGTATAAGAGACAGGARTGYCCDGGDCAYTTYGG  GTCTCGTGGGCTCGGAGATGTGTATAAGAGACAGCCNGCDATNTCRTTRTCCATRTA | [4, 5] |
| RPB2_F1  RPB2_R1 | TCGTCGGCAGCGTCAGATGTGTATAAGAGACAGGAYGAYMGWGATCAYTTYG  GTCTCGTGGGCTCGGAGATGTGTATAAGAGACAGGCAGGRCARACCAWMCCCCA | [6] |
| RPB2_F2  RPB2_R2 | TCGTCGGCAGCGTCAGATGTGTATAAGAGACAGTGGGGKWTGGTYTGYCCTGC  GTCTCGTGGGCTCGGAGATGTGTATAAGAGACAGCCCATRGCTTGYTTRCCCAT | [6] |
| MCM7_F  MCM7_R | TCGTCGGCAGCGTCAGATGTGTATAAGAGACAGACIMGIGTITCVGAYGTHAARCC  GTCTCGTGGGCTCGGAGATGTGTATAAGAGACAGGAYTTDGCIACICCIGGRTCWCCCAT | [7] |

*Note:* Overhang Illumina adapter sequences are marked with blue and orange; sequences that are necessary for amplification of target regions are marked with black. The references correspond to the parts of primers marked with black. Two primer pairs for the *RPB2* gene were used to amplify two regions of this gene. I – inosine.

**IUPAC nucleotide codes**

| **Code** | R | Y | S | W | K | M | B | D | H | V | N |
| --- | --- | --- | --- | --- | --- | --- | --- | --- | --- | --- | --- |
| **Base** | A or G | C or T | G or C | A or T | G or T | A or C | C or G or T | A or G or T | A or C or T | A or C or G | any base |

**References**

[1] T.J. White, T. Bruns, S. Lee, J.W. Taylor, Amplification and direct sequencing of fungal ribosomal RNA genes for phylogenetics, in: M.A. Innis, D.H. Gelfand, J.J. Sninsky, T.J. White (Eds.), PCR Protocols: A Guide to Methods and Applications, Academic Press, Inc., New York, 1990, pp. 315-322.

[2] N.L. Glass, G.C. Donaldson, Development of primer sets designed for use with the PCR to amplify conserved genes from filamentous ascomycetes, Appl Environ Microbiol. 61 (1995) 1323-1330.

[3] J.B. Stielow, C.A. Levesque, K.A. Seifert, W. Meyer, L. Iriny, D. Smits, R. Renfurm, G.J. Verkley, M. Groenewald, D. Chaduli, A. Lomascolo, S. Welti, L. Lesage-Meessen, A. Favel, A.M. Al-Hatmi, U. Damm, N. Yilmaz, J. Houbraken, L. Lombard, W. Quaedvlieg, M. Binder, L.A. Vaas, D. Vu, A. Yurkov, D. Begerow, O. Roehl, M. Guerreiro, A. Fonseca, K. Samerpitak, A.D. van Diepeningen, S. Dolatabadi, L.F. Moreno, S. Casaregola, S. Mallet, N. Jacques, L. Roscini, E. Egidi, C. Bizet, D. Garcia-Hermoso, M.P. Martin, S. Deng, J.Z. Groenewald, T. Boekhout, Z.W. de Beer, I. Barnes, T.A. Duong, M.J. Wingfield, G.S. de Hoog, P.W. Crous, C.T. Lewis, S. Hambleton, T.A. Moussa, H.S. Al-Zahrani, O.A. Almaghrabi, G. Louis-Seize, R. Assabgui, W. McCormick, G. Omer, K. Dukik, G. Cardinali, U. Eberhardt, M. de Vries, V. Robert, One fungus, which genes? Development and assessment of universal primers for potential secondary fungal DNA barcodes, Persoonia. 35 (2015) 242-263. 10.3767/003158515X689135.

[4] J.W. Stiller, B.D. Hall, The origin of red algae: implications for plastid evolution, Proc Natl Acad Sci U S A. 94 (1997) 4520-4525. 10.1073/pnas.94.9.4520.

[5] P.B. Matheny, Y.J. Liu, J.F. Ammirati, B.D. Hall, Using RPB1 sequences to improve phylogenetic inference among mushrooms (Inocybe, Agaricales), Am J Bot. 89 (2002) 688-698. 10.3732/ajb.89.4.688.

[6] Y.J. Liu, S. Whelen, B.D. Hall, Phylogenetic relationships among ascomycetes: evidence from an RNA polymerse II subunit, Mol Biol Evol. 16 (1999) 1799-1808. 10.1093/oxfordjournals.molbev.a026092.

[7] I. Schmitt, A. Crespo, P.K. Divakar, J.D. Fankhauser, E. Herman-Sackett, K. Kalb, M.P. Nelsen, N.A. Nelson, E. Rivas-Plata, A.D. Shimp, T. Widhelm, H.T. Lumbsch, New primers for promising single-copy genes in fungal phylogenetics and systematics, Persoonia. 23 (2009) 35-40. 10.3767/003158509X470602.
